# Target-Target Perceptual Similarity Within the Attentional Blink

**DOI:** 10.3389/fpsyg.2020.551890

**Published:** 2020-12-16

**Authors:** Ivan M. Makarov, Elena S. Gorbunova

**Affiliations:** School of Psychology, National Research University Higher School of Economics, Moscow, Russia

**Keywords:** task relevance, interference, perceptual similarity, attentional blink, visual attention

## Abstract

Three experiments investigated the role of target-target perceptual similarity within the attentional blink (AB). Various geometric shapes were presented in a rapid serial visual presentation task. Targets could have 2, 1, or 0 shared features. Features included shape and size. The second target was presented after five or six different lags after the first target. The task was to detect both targets on each trial. Second-target report accuracy was increased by target-target similarity. This modulation was observed more for mixed-trial design as compared with blocked design. Results are discussed in terms of increased stability of working memory representations and reduced interference for second-target processing.

## Introduction

Attentional blink (AB) is the deficit in detection and/or discrimination of the second-target stimulus on correct report of the first target, when the targets are presented in a short temporal succession (Raymond et al., 1992). In rapid serial visual presentation (RSVP) tasks, participants have to detect two target stimuli presented sequentially in the center of a screen. In a typical display, 10 stimuli/s are displayed. The attentional blink usually occurs if the second target is presented between 200 and 500 ms after the first target. The attentional blink occurs with different types of stimuli: letters (Raymond et al., 1992), words (Wierda et al., 2013), more complex objects like faces (Sy and Giesbrecht, 2009), as well as colors (Ross and Jolicoeur, 1999), scenes (Einhäuser et al., 2007), sounds (Horváth and Burgyán, 2011), and touch (Soto-Faraco et al., 2002).

Several theories have been introduced to explain the AB. The explanation of second-target omission was related to inhibition, interference, bottleneck, or temporal loss of control (see Dux and Marois, 2009). Also, AB theories can be divided in two groups: T1-based theories, assuming that T1 processing is sufficient to produce the blink, and distractor-based theories, in which the presence of at least one distractor following T1 is essential (Lagroix et al., 2012).

Bottleneck theories assume that AB results primarily from a central bottleneck of information processing. One of the first bottleneck assumptions was made by Chun and Potter (1995), who proposed the capacity-limited stage of informational processing as the reason of AB. Another example is the attentional dwell time hypothesis, proposed by Ward et al. (1996), which also assumes two targets to compete for capacity-limited resources, with the “winner” of this competition undergoing later processing at the expense of the “loser.” Because of its head start in the competition, Tl is typically the winner, and T2 is likely to go undetected.

According to the inhibition theory (Raymond et al., 1992), the AB is observed due to the identification process of the first target. In RSVP tasks, in order to reduce target-distractor feature confusion, the “attentional gate” stays closed until the identification of the first target is complete. This process takes approximately 500 ms, so the second target is not recognized if it is presented in this time course. However, no attentional blink was observed for emotionally relevant stimuli (Anderson and Phelps, 2001). Also, the subject was able to recognize the second-target stimuli if it was his or her personal name (Shapiro et al., 1997b).

According to interference theory (Shapiro et al., 1994), in the RSVP paradigm, the initial perceptual representations are created for each target or distractor. These representations are compared with target templates, and those stimuli that match the templates are selected and proceed to working memory. In working memory, the interference occurs as the retrieval process is undertaken during the report of the targets. The AB happens because each target receives a weight based on its similarity to the template and the available working memory capacity, and the latter is limited when the targets are displayed in short temporal succession.

A two-stage model proposed by Chun and Potter (1995) has some similar aspects to the interference theory. The difference is related to processes in working memory. According to the two-stage model, the process of target identification in RSVP tasks has two stages. At the first stage, rapid recognition occurs as the stimulus activates a stored conceptual representation in the visual system. At the second stage, the stimulus is encoded to working memory, and this stage has capacity limitations. The AB occurs when the probe is presented in temporal proximity to the first target, because the probe has to “wait” to be encoded into working memory after the first target has been fully processed. Therefore, the second target has more distractor interruption and is decay prone. A modification of this theory is known as the three-stage model (Peterson and Juola, 2000) and has an additional stage of detecting the spatial arrangement of stimuli.

Overall, all these models of attentional blink assume the role of target selection, resource competition, or both. Targets compete for resource-limited processing stages, which are based on selection mechanisms. However, T1-capacity-limited models are challenged by the results of Di Lollo et al. (2005). They presented subjects with RSVP streams that contained three successive targets, all of which required reporting. The third target was displayed in a position where the blink is typically maximal (Lag 2). An impaired third target report was found if the second target belonged to a category different from that of the other stimuli, whereas no deficit in reporting the third target was observed when the targets belonged to the same category. Later studies revealed reduced AB when two targets were words in sentences (Potter et al., 2008) or letters in words (Falikman, 2002; Stepanov, 2009). According to the temporary loss of control hypothesis (Di Lollo et al., 2005), RSVP processing is operated by a filter configured to select targets. This filter is under the endogenous control of a central processor that can run only one operation at a time. When a target is initially identified, the central processor switches from monitoring to consolidation processes, and the filter undergoes exogenous control. When the second target belongs to the same category as the first one, the filter’s configuration does not have to be altered, so this target is processed efficiently. When the second target belongs to a different category, the filter’s configuration is disrupted and needs to be reconfigured, resulting in less-efficient processing in the subsequent stimuli.

The temporary loss of control hypothesis would predict the second target would be processed more efficiently when there is high target-target similarity, because there is no need for the filter to be altered. This account is supported by studies of priming. An improved recall of the second-target word was observed in the AB interval when the second target was a strong associate of either the first target or a distractor occurring in the AB interval (Maki et al., 1997). Moreover, letter targets missed through the AB, enhanced the performance for subsequent targets if they had a match in identity, as well as semantically related words (Shapiro et al., 1997a). Another point for the temporary loss of control account is that the magnitude of the AB is greater when the intertarget interval contains distractors than when it is blank (see Lagroix et al., 2012, for discussion). However, more recent studies discovered that the only role of distractors in modulating the magnitude of the blink is through the backward masking of the first target but not through the disruption of the input control.

A direct comparison of semantic and repetition priming was made by Pesciarelli et al. (2007). Three target words were presented in a stream of nonword distractors. The first word was not related to either the second or third word. The second and third words could be unrelated words, semantically related words, or identical, in order to test both repetition and semantic priming effects. Both semantic and repetition priming effects were observed in the third-word accuracy (in behavioral and neural measures), whether the second target was reported or missed.

Davenport and Potter (2005) tested the locus of semantic priming in an RSVP target search. The word associated with one or neither of the two target words was presented at the beginning of each trial. When both targets were to be reported, semantically primed targets were reported with greater accuracy than were unprimed targets. In the additional experiment, when only the primed word was reported, accuracy was at the same level as for semantically primed targets in the first experiment. There was also a greater benefit for the primed target at short SOAs, meaning that priming actually biases the competition in favor of the primed target and against the unprimed target at stage 1, according to the two-stage competition model. So, the effect of semantic priming seems to facilitate the lexical identification of one target while the two targets are in competition. In the latter experiments, Potter et al. (2005) varied the semantic relatedness between the targets to discover if T2 is able to be identified before T1 at different SOAs. When the targets were semantically related, both showed a priming benefit at short SOAs (less than 100 ms), and only T2 benefited from priming at longer SOAs. Meaning that T2 is able to be identified before T1 at short SOAs (so bidirectional priming is possible on SOAs less than 100 ms), but not on longer SOAs (only unidirectional priming is possible).

The magnitude of the AB was also shown to increase when T1 and T2 were assumed to be from different categories. In the Taylor and Hamm (1997) experiment, the target/probe categorical relation was manipulated by the instructional set: the target “O,” presented among letters, was referred to as the letter “oh” or as the number “zero.” Treating O as a number attenuated the probe detection deficit. However, Ward et al. (1997) did not reveal any effect of similarity when T2 was a letter, and T1 was a letter or an outline box. The task for T1 required subjects to discriminate between two possible sizes, whereas the task for T2 was to determine whether the second target was the letter X. One of the possible explanations for that result is that T1 identity was not relevant to the task and participants never encoded its identity. Another explanation might come from the paradigm used in this study: a skeletal RSVP stream consisting of only two targets and their respective pattern masks could minimize the demands on selective attentional processing.

An alternative account is proposed by the over-investment hypothesis (Olivers and Nieuwenhuis, 2006). According to this hypothesis, too many attentional resources are devoted to the first target encoding, which results in the inadvertent processing of distractors which then impair the second target accuracy. This theory is consistent with the results of Visser et al. (2009), who revealed the role of similarity in Lag 1 sparing. According to their data, high target-distractor similarity (targets: letters; distractors: digits as compared with targets: letters, distractors: random-dot patches) improves probe detection accuracy when the targets follow one another directly, and impairs it at all subsequent lags. On Lag 1, both targets are processed simultaneously, so over-committal of resources to the first-target leaves extra resources available for the processing of the second target. When the target-distractor similarity (and the difficulty of the task) is increased, accuracy at Lag 1 is increased. However, MacLellan et al. (2018) have shown that the over-investment might be limited by the perceptual mechanisms that evaluate the need for encoding resources. Manipulation of target predictability using a mixed-trial or blocked design, revealed that even a relatively easy to encode T1 caused an attentional blink when it was perceptually similar to a more difficult to encode T1.

Whereas most of the studies in attentional blink are interested in target-distractor similarity (due to its relevance to test assumptions about the target selection), the similarity between the first and second target was also shown to modulate the attentional blink. For example, the deficit was more severe when both targets demanded the same type of processing (Awh et al., 2004). In particular, a T1 digit discrimination task induced long-lasting attentional blink interference for subsequent letter discrimination but did not disrupt face discrimination. Authors suggest that faces avoid interference because face discrimination is related to the configural processing channel which was not disrupted by the T1 digit task, related to featural processing. According to the multiple-resource channel hypothesis (Awh et al., 2004), the severity of attentional blink is related to T1 and T2 processing mechanisms: as the processing mechanisms required for T2 increase in overlap with those required for T1, the severity of the attentional blink would also increase.

The results of Sy and Giesbrecht (2009) experiment revealed the role of task relevance in target similarity. The stimuli included faces with different emotional expressions. Participants indicated the gender of T1 and T2 at the end of each trial. Similarity between target faces was manipulated in two dimensions, one of which was task relevant (gender) and the other was not (emotional expression). The results indicated that similarity of the task-relevant dimension modulated the attentional blink, while similarity on the task-irrelevant dimension did not.

Although in attentional blink studies described above, target-target similarity was usually assumed to induce more target interference, there are studies in other paradigms that revealed the opposite effect. In the Lin and Luck (2009) study, interference in working memory was studied through the change detection paradigm. It was found that the level of interference decreased when increasing the similarity in color of the stimuli. This result is explained by the increased stability and accuracy of representation in memory. A recent study conducted in the dual-target visual search paradigm (Gorbunova, 2017) also revealed the role of perceptual target-target similarity: the second target accuracy increased when increasing the number of shared features (color, shape, and size) between T1 and T2.

Another phenomenon similar to AB that should be addressed here is repetition blindness (RB) – a deficit of reporting the second repeated stimulus in RSVP stream (Kanwisher, 1987). Despite the similarity between AB and RB, these phenomena differ significantly in the paradigm: RB refers to reporting the repetitions of targets, whereas AB occurs for unrepeated targets. Also, RB and AB follow different time courses: AB occurs on particular lag, whereas RB magnitude typically decreases as a function of increasing lag (e.g., Park and Kanwisher, 1994). Moreover, a double dissociation was observed between AB and RB (Chun, 1997). Increased target-distractor discriminability alleviated AB but not RB, whereas enhanced episodic distinctiveness of the two targets (presenting T1 and T2 in different colors) eliminated RB but not AB.

One of the possible explanations for differences in results observed for different AB studies is the type of target similarity. Whereas some AB studies manipulated the complex types of similarity (type of processing), AB studies on priming and categorical similarity, as well as visual search and working memory studies, used the simple perceptual difference (lexical, categorical, basic features). Therefore, the similarity of the task level may increase the number of resources required for T2 processing, and those increase the AB magnitude, whereas similarity on the level of target type might have the opposite effect, due to the increased stability of working memory representations.

In the current study, we were going to address this issue. The manipulation assumed here refers to the level of basic features, assuming both task-relevant and task-irrelevant target similarity. The two-stage model would assume that an increase in target perceptual similarity under the conditions of RVSP is expected to increase the stability of working memory representations and thereby reduce interference. So, the magnitude of the “attentional blink” is expected to decrease with the increasing number of shared features in the first- and second-target stimuli, and T2 detection is expected to increase inside the AB interval.

## Experiment 1

### Method

#### Participants

Twenty-five students of the National Research University Higher School of Economics: 19 females and 6 males volunteered for class credit. The age varied from 18 to 21 years old (M = 18.72, SD = 0.98). All participants were native Russian speakers, naive to experimental hypotheses, and reported normal or corrected-to-normal visual acuity. Informed consent was obtained from all subjects.

#### Stimuli

Stimuli varied in shape, size and color. Overall, there were 24 different types of stimuli. Geometric shapes of squares, triangles, circles and pentagons were used. There were two types of figures: small (1.72 × 1.88°) and big (2.23 × 2.43°). Both targets and distractors could be small or big. The color was different for targets and distractors. The first target was always yellow. The second target was always blue. All distractors were green. Stimuli used for participant’s responses were black and were medium sized (1.98 × 2.08°), because participants had to indicate only the correct form of target. At the beginning of each trial, a black fixation cross (size: 1.15 × 1.25°) was presented at the center of the screen. After each trial a mask (a random noise picture) was displayed at the center of the screen (size: 2.87 × 3.13°). All stimuli were presented on a gray background.

Participants sat in a dark room 60 cm from a 21.5 in. BENQ-GL2250 monitor (screen resolution: 1920 × 1,080; refresh rate: 60 Hz). Stimuli were displayed with Psychopy v. 1.84.01, OS Windows 7. Participant’s responses were registered with a standard keyboard.

Two factors were manipulated in this experiment. First, the target’s perceptual similarity was varied by the number of shared features between T1 and T2 (shape and size). There were three conditions: two shared features (T1 and T2 had the same shape and size), one shared feature (T1 and T2 had the same shape or the same size and differed by another feature), and 0 shared features (T1 and T2 had different shape and size). Second, the T1-T2 lag was manipulated such that T2 could be presented on the first lag – right after the T1, on second, third, fourth, or fifth lag after the T1.

#### Procedure

The experiment consisted of 320 trials. Forty trials were conducted for the condition with two shared features for T1 and T2 (e.g., T1 – big square, T2 – big square). One hundred twenty trials were conducted for the condition with equal size for T1 and T2 (e.g., T1, big square; T2, big triangle). Forty trials were conducted for the condition with equal shape for T1 and T2 (e.g., T1, big circle; T2, small circle). One hundred twenty trials were conducted for the condition with 0 shared features for T1 and T2 (e.g., T1, big pentagon; T2, small triangle).^1^ At the beginning of each trial, the fixation cross was presented for 500 ms in the center of the screen. After that, 14 figures were displayed sequentially in the center of the screen (duration of each presentation was 99.6 ms), each was followed by 16.6 ms ISI.^2^ At the end of each trial, a mask was presented for 116.6 ms. From two to eight distractors were presented before T1 appeared; distractors never had the same identity as the targets. The second target was presented on five different lags after the first target. The order of presentation was randomized. The mask was presented at the end of each trial in order to destroy the sensory trace of the last presented stimulus.

The participants’ task was to indicate the shape of T1 and T2 at the end of each trial. Two alternative forced choice (2AFC) procedures were used, in order to make the results comparable with other AB studies, e.g., Sy and Gisbrecht (2009), that used 2AFC procedure. Participants had to choose the correct response between two black figures of medium size – first for T1, then for T2. When the target was displayed on the right, they pressed the right arrow key. When the target was displayed on the left, they pressed the left arrow key.

**Figure 1:**
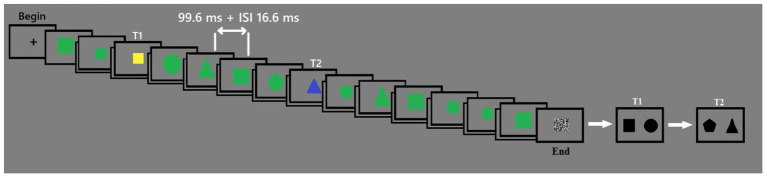
The timeline of the experiment. Each stimulus was presented for 99.6 ms. ISI was 16.6 ms. The first target was yellow, and the second target was blue. Distracters were green.

A training session of 10 trials preceded the experiment. The example of the trial design is shown in Figure 1.

### Results

#### T1 Performance

The first-target report accuracy was analyzed. Repeated measures ANOVA was used, the factors included lag (levels: lags 1, 2, 3, 4, and 5) and the number of shared features (levels: two shared features, one shared feature, and 0 shared features). Pairwise comparisons were made with Bonfferroni corrections; corrections were applied within each analysis. Greenhouse-Geisser corrections were applied for significant Mauchy’s test results. Data analysis was conducted using IBM SPSS Statistics 23.

ANOVA revealed the significant effect of the lag factor [*F* (1, 34) = 8.2, *p* = 0.003, *ηp*^2^ = 0.255] and of the number of shared-feature factor [*F* (3, 64) = 13.04, *p* < 0.001, *ηp*^2^ = 0.352]. The interaction was also significant [*F* (4, 89) = 5.93, *p* < 0.001, *ηp*^2^ = 0.198].

A separate ANOVA of lag factor (levels: lags 1, 2, 3, 4, and 5) was conducted within each level of the shared-feature factor due to significant interaction. ANOVA for the 0 shared-feature condition revealed no significant effect of lag factor [*F* (4, 96) = 1.43, *p* = 0.229, *ηp*^2^ = 0.056]. For the one shared-feature condition, a significant effect of the lag factor was observed [*F* (4, 96) = 8.745, *p* < 0.001, *ηp*^2^ = 0.267]. Lag 2 accuracy was higher as compared with lag 1 (*t* = 1.287, *p* = 0.004, *d* = 0.272) and lower as compared with lag 4 (*t* = −0.495, *p* = 0.009, *d* = −0.135) and lag 5 (*t* = −0.825, *p* < 0.001, *d* = −0.201).

For the two shared-feature condition, the significant effect of the lag factor was also present [*F* (3, 61) = 8.865, *p* < 0.001, *ηp*^2^ = 0.270]. Lag 2 accuracy was higher compared with lag 4 (*t* = 3.266, *p* = 0.033, *d* = 0.754) and lag 5 (*t* = 4.218, *p* = 0.001, *d* = 1.475), and lag 3 accuracy was higher than lag 4 (*t* = 4.919, *p* < 0.001, *d* = 0.227).

**Figure 2:**
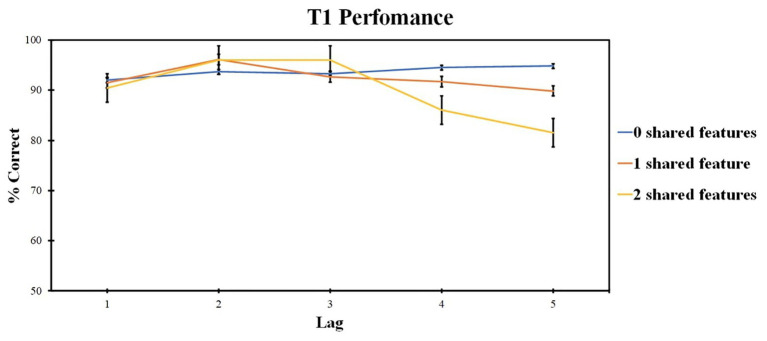
Results of Experiment 1: T1 identification accuracy. Error bars show the standard errors of the mean.

Those results are presented in Figure 2.

#### T2|T1 Performance

The second-target report accuracy was analyzed if there was a correct T1 detection (T2|T1). Repeated measures ANOVA was used; the factors included lag (levels: lags 1, 2, 3, 4, and 5) and the number of shared features (levels: two shared features, one shared feature, and 0 shared features). Pairwise comparisons were made with Bonfferroni corrections, corrections were applied within each analysis. Greenhouse-Geisser corrections were applied for significant Mauchy’s test results. Data analysis was conducted using IBM SPSS Statistics 23. Data were also examined with Bayesian repeated measures ANOVA in JASP. All Bayes factors reported were estimated using the default prior parameters.

**Figure 3:**
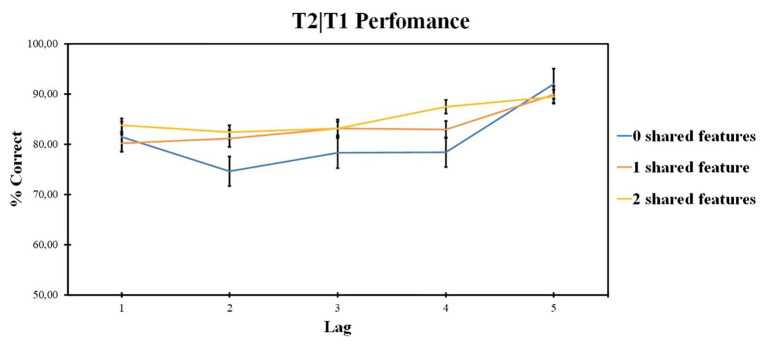
Results of Experiment 1: T2 identification accuracy for trials on which T1 was correctly identified (T2|T1). Error bars show the standard errors of the mean.

ANOVA revealed the significant effect of the lag factor [*F* (2, 48) = 4.85, *p* = 0.012, *ηp*^2^ = 0.168, BF_10_ = 144.863] and of the number of shared-feature factor [*F* (4, 96) = 10.33, *p* < 0.001, *ηp*^2^ = 0.301, BF_10_ = 1.499]. The interaction was not significant [*F* (4, 99) = 1.39, *p* = 0.243, *ηp*^2^ = 0.055]. The results are presented in Figure 3.

According to pairwise comparisons for different levels of the shared-feature factor, 0 shared-feature condition accuracy was lower as compared with two shared-feature condition (*t* = 0.262, *p* < 0.020, *d* = 0.049) and lower as compared with one shared-feature condition (*t* = −1.146, *p* = 0.008, *d* = −0.149). Overall, lag 5 accuracy was higher as compared with lag 1 (*t* = 6.238, *p* < 0.001, *d* = 1.11), lag 2 (*t* = 6.027, *p* < 0.001, *d* = 1.194), lag 3 (*t* = 3.391, *p* < 0.001, *d* = 0.595), and lag 4 (*t* = 2.807, *p* < 0.001, *d* = 0.663). Other differences are not significant, *p* > 0.05.

**Figure 4:**
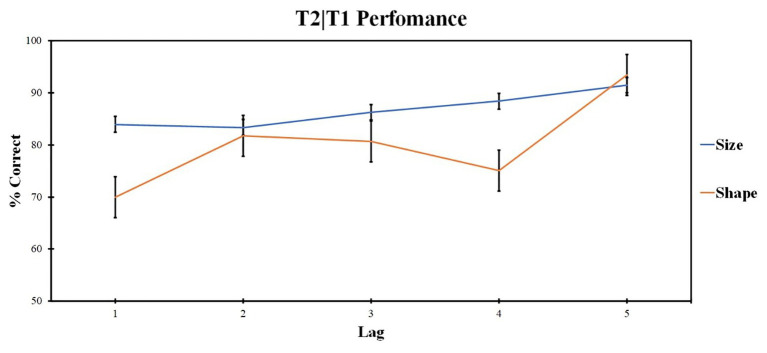
T2|T1 accuracy for the one shared-feature condition for the shape and size feature condition (Experiment 1). Results are displayed for T2 identification in trials when T1 was correctly identified (T2|T1). Error bars represent the standard errors of the mean.

Additional *post hoc* analysis on the influence of shape and size features for the one shared-feature condition was made to understand the impact of each feature similarity on T2 accuracy. Two-way ANOVA compared the accuracy for different feature factors (levels: same shape, different size condition and same size, different shape condition) at different lags (levels: lags 1, 2, 3, 4, and 5). Repeated measures ANOVA revealed the significant impact of the lag factor [*F* (4, 96) = 9.87; *p* < 0.001; *ηp*^2^ = 0.291] and the feature factor [*F* (1, 24) = 7.08; *p* = 0.014; *ηp*^2^ = 0.228]. The interaction is also significant [*F* (3, 76) = 3.335; *p* = 0.022; *ηp*^2^ = 0.122]. The results are presented in Figure 4.

Pairwise comparisons were conducted for different levels of lag factors within different levels of feature factors and vice versa. Pairwise comparisons for feature factor revealed significant differences in the first lag; lag 1 accuracy in shared size feature condition was higher as compared with lag 1 accuracy in shape shared-feature condition (*t* = −1.96, *p* = 0.008, *d* = 0.903). Lag 4 accuracy in size shared-feature condition was higher as compared with lag 4 accuracy in shared shape feature condition (*t* = −1.532, *p* = 0.022, *d* = 1.054).

For shared size feature condition, lag 5 accuracy was higher as compared with lag 1 (*t* = 3.165, *p* = 0.044, *d* = 0.795), lag 2 (*t* = 3.587, *p* = 0.001, *d* = 0.874), and lag 3 (*t* = 2.727, *p* = 0.009, *d* = 0.734). Lag 2 accuracy was lower as compared with lag 4 (*t* = −2.426, *p* = 0.024, *d* = −0.542).

For shared shape feature condition, lag 5 accuracy was higher as compared with lag 1 (*t* = 6.466, *p* < 0.001, *d* = 1.624), lag 2 (*t* = 5.331, *p* < 0.001, *d* = 1.076), lag 3 (*t* = 3.683, *p* = 0.001, *d* = 0.971), and lag 4 (*t* = 5.735, *p* < 0.001, *d* = 1.423).

As a difference between shape and size features for the one shared-feature condition was revealed, another analysis was conducted in order to understand the influence of posttarget distracters on target detection accuracy (for example, big-sized targets could be not fully masked by small-sized subsequent distracters). One-way ANOVA for T1 accuracy in 0 shared features was conducted (this condition was chosen because no lag effects were revealed), levels included a big target masked a small distracter, a big target masked by a big distracter, a small target masked a small distracter, and a small target masked by a big distracter. No significant effect of subsequent distracter type was revealed [*F* (1, 24) = 3.38, *p* = 0.08, *ηp*^2^ = 0.12].

Those results are presented in Figure 4.

### Discussion

The role of targets perceptual similarity within the AB interval was revealed in this experiment. Accuracy for second target detection increased when the number of shared features between two targets increased. A possible explanation might be the increased stability of working memory representations and therefore reduced interference. However, no interaction between lag and targets similarity was observed, so, according to the data, target similarity produced a general increase of T2 efficiency, but not a specific AB magnitude reduction.

For the one shared-feature condition, a difference in accuracy for shape and size features was observed, with similarity in the shape feature producing a severe AB deficit. Similarity in the size feature (task-irrelevant) revealed a classical AB with lag 1 sparing and gradual recovery from the blink, whereas similarity in shape condition (task-relevant) revealed no lag 1 sparing and no gradual recovery. This might be related to the differences in shape and size feature processing, or to the differences in task-relevant (shape) and task-irrelevant (size) feature processing. No significant differences between two shared features and one shared feature were observed. Taken together, these results might assume that similarity in task-relevant features would produce a severe attentional blink deficit, whereas similarity in task-irrelevant features would enhance the T2 performance. These results are related both to the results of Sy and Giersbrecht (2009) experiment, in which accuracy was greater when the targets were similar on the task-relevant dimension, and to the results of priming studies (e.g., Maki et al., 1997), when the T1 and T2 similarity enhanced the T2 performance.

Regarding the specific lag 1 difference, according to Visser et al. (1999), no lag 1 sparing was observed when the attentional switch in location was required. From that point, dissimilarity in size in T1 and T2 in our experiments might be observed as a spatial shift of attention (more precisely, in the spatial variation of the attentional focus extension), thus leading to lag 1 sparing.

The results of Dell’Acqua et al. (2007) revealed the lag 1 sparing effect in a digit identification task, when the participant had to report the identity of target digits, but not in a counting task, when the observer had to count how many digits were presented. Lag 1 sparing was found when individual character identities were required by the task and was absent when the task could be performed on the basis of less specific category-level information. According to the central interference theory (Jolicoeur and Dell’Acqua, 1998), lag 1 effect is caused by two targets entering the same short-term consolidation batch and consolidated simultaneously. Categorizing the target as a target (like in the counting task) is likely to take less time than deciding exactly which character had been presented (like in the identification task). From this point of view, postselection capacity-demanding operations would start sooner in the counting task when compared with the identification task. As a result of this, the probability of the second target to be included in the same consolidation batch as the first target would be higher when consolidation initiates later (in the identification task) as compared with a more prompt consolidation (in the classification task). In relation to our experiment, if both targets proceed to the same consolidation batch (as in the identification task), the interference might be observed, especially when task-relevant feature similarity (when T1 has the same shape as T2), thus causing the decrease at lag 1.

Another explanation might come from the over-investment hypothesis (Olivers and Nieuwenhuis, 2006) and from the results of a study by Visser et al. (2009), where high target-distractor similarity improved probe detection accuracy at lag 1. Target-distractor similarity increased the difficulty of the task, thus increasing the number of resources available for the second-target processing and probe report accuracy at lag 1. In our experiments, when the target-target similarity was increased in the one shared-feature condition, the task difficulty was decreased more for task-relevant feature (shape) as compared with task-irrelevant feature (size). The decrease in the resources devoted to target processing was more severe for the shape condition compared with the size condition, so the accuracy at lag 1 was decreased more in the shape condition compared with the size condition.

One of the points that should be taken into account is T1 accuracy. T1 accuracy was not affected by lag for the 0 shared-feature condition, whereas the significant decrease in T1 accuracy on lags 4 and 5 was observed for the one shared-feature condition and for the two shared-feature condition. At this end, T1 and T2 similarity leads to decreased performance of T1 on the later lags (and for lag 1 for one shared-feature condition). There are three possible ways how the T1-T2 similarity can decrease on T1 performance: by masking the T1 representation, by disrupting the sequence of target perception, or by the impact of participants’ reported strategy. As the decrease of T1 accuracy is observed on the interval of recovery from the blink (and on lag 1), when T2|T1 accuracy is increased, and no resource processing deficit should occur, the first explanation seems to be unlikely. Also, T2 should not be identified before T1 at late SOAs (Potter et al., 2005), so this result is unlikely to be related to the sequence of target perception as well, so the probable explanation is related to the participants’ reported strategy. On lags 4 and 5, the participants do not always correctly identify T1, but if T1 is identified correctly, T2 is identified correctly too. For example, if the targets have the same shape (as they do in half trials of the one shared-feature condition and all trials in the two shared-feature condition), and the participant firstly gives a response about T1, there is the possibility of confusion. However, the question remains, why does T1 accuracy decrease on lags 4 and 5, but not on lags 2 and 3 – inside the “blink” interval. The explanation might be related to better T1 and T2 processing in WM inside the blink interval to target similarity, that, in turn, would impact on participants’ response confidence.

Another point here is that the design of Experiment 1 did not properly balance the frequency of task-relevant conditions. The difference in the frequencies might produce a response bias. Regarding this point, another experiment was conducted in order to balance the conditions.

## Experiment 2

### Method

#### Participants

Twenty-two students of the HSE University, 12 females and 10 males volunteered for class credit. The age varied from 18 to 24 years old (M = 20.13, SD = 1.59). All participants were native Russian speakers, naive to the experimental hypothesis, and reported normal or corrected-to-normal visual acuity. Informed consent was obtained from all subjects.

#### Stimuli and Procedure

Stimuli and procedure were the same as in Experiment 1, except for the ratio of trials for different task-relevant conditions. Overall, there were 480 trials, distributed equally between five lag conditions and between four shared-feature factor conditions as follows: two shared features (shape and size), shared size, shared shape, and 0 shared features.

### Results

Data analysis followed the analysis in Experiment 1, except the separation of the same shape and same size conditions. The factors included the number of shared features (levels: two shared features, same shape, same size, 0 shared features) and lag (lags 1–5). Moreover, a separate ANOVA for lag factor (levels: lags 1, 2, 3, 4, and 5) was conducted within each level of the shared-feature factor in order to precisely test the AB effect for each condition (due to the significant lag × shared-feature interaction). Pairwise comparisons were made with Bonferroni corrections; corrections were applied within each analysis.

#### T1 Performance

ANOVA revealed the significant effect of the number of shared-feature factor [*F* (2, 46) = 4.41, *p* = 0.015, *ηp*^2^ = 0.174] and of the lag factor [*F* (4, 84) = 5.51, *p* = 0.001, *ηp*^2^ = 0.208]. The interaction was not significant [*F* (7, 152) = 1.83, *p* = 0.084, *ηp*^2^ = 0.080]. Pairwise comparisons did not reveal any significant differences between the levels of the number of shared-feature factor. Lag 2 accuracy was higher as compared with lag 4 (*t* = 3.649, *p* = 0.008, *d* = 0.142) and lag 5 (*t* = 4.874, *p* < 0.001, *d* = 0.189).

**Figure 5:**
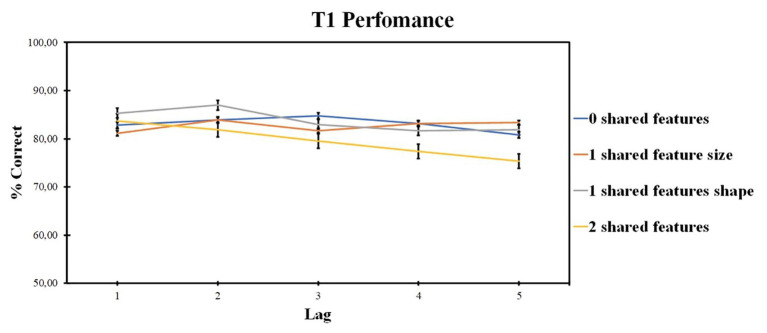
Results of experiment 2: T1 identification accuracy. Error bars show the standard errors of the mean.

Those results are presented in Figure 5.

#### T2|T1 Performance

ANOVA revealed no significant effect for the number of shared-feature factor [*F* (2, 40) = 2.27, *p* = 0.089, *ηp*^2^ = 0.098, BF_10_ = 5.853]. The lag factor was significant [*F* (4, 84) = 5.49, *p* = 0.001, *ηp*^2^ = 0.207, BF_10_ = 76.064]. The interaction was also significant [*F* (12, 252) = 2.95, *p* = 0.001, *ηp*^2^ = 0.123].

ANOVA within the 0 shared-feature condition revealed the significant impact of the lag factor [*F* (4, 84) = 8.92; *p* < 0.001; *ηp*^2^ = 0.298]. According to pairwise comparisons, lag 1 accuracy was higher as compared with lag 2 (*t* = 3.252, *p* = 0.012, *d* = 0.478), lag 5 accuracy was higher as compared with lag 2 (*t* = 2.401, *p* < 0.001, *d* = 0.669), lag 3 (*t* = 4.535, *p* = 0.019, *d* = 0.306), and lag 4 (*t* = 3.649, *p* < 0.001, *d* = 0.486).

ANOVA within the shared size feature condition revealed no significant impact of lag factor [*F* (4, 84) = 1.95; *p* = 0.109; *ηp*^2^ = 0.085].

ANOVA within the shared shape feature condition revealed the significant impact of lag factor [*F* (3, 65) = 4.56; *p* = 0.005; *ηp*^2^ = 0.179]. According to pairwise comparisons, lag 1 accuracy was lower as compared with lag 5 (*t* = −2.492, *p* = 0.006, *d* = 0.445).

ANOVA within the two shared-feature condition revealed no significant impact of the lag factor [*F* (3, 59) = 1.4; *p* = 0.252; *ηp*^2^ = 0.063].

**Figure 6:**
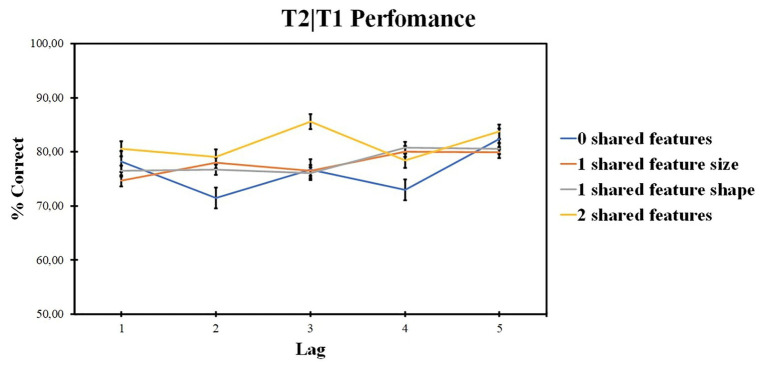
Results of experiment 2: T2 identification accuracy for trials on which T1 was correctly identified (T2|T1). Error bars show the standard errors of the mean.

Those results are presented in Figure 6.

### Discussion

In this experiment, T1 accuracy gradually decreased both with T1-T2 similarity and with lag; however, no interaction was observed, and pairwise comparisons did not reveal any significant effects for similarity. Basically, T1 performance at lag 2 is better as compared with lags 4 and 5, whereas T2 performance was decreased for early lags. This result is consistent with the pattern observed by Potter et al. (2002), when the competition of two subsequently presented words and various SOAs was observed. At SOAs in the range of 13–53 ms, the second presented word was more likely to be reported, but at 213 ms, the advantage switched to the first word, as in the attentional blink. Visser et al. (2009) assumes that accuracy for both T1 and T2 varies dynamically as a function of intertarget interval. However, no interaction of the similarity and lag factors was observed for T1 accuracy, so the T2 results should be independent from the T1 detection difference.

For T2 accuracy, a significant influence of both lag and number of shared-feature factors was observed, as well as the significant interaction between the lag and number of shared-feature factors. Firstly, T2 accuracy increased with an increase in target similarity. The classical AB pattern – a decrease in accuracy on positions 2–4 with lag 1 sparing and recovery on lag 5 was observed for the 0 shared-feature condition. No effect of lag was observed for the shared size feature condition and for the two shared-feature condition. T2 accuracy gradually increased with the lag in shared shape feature condition.

The increase of T2 accuracy in two shared-feature condition and in shared size feature condition is consistent with our initial predictions: AB magnitude was reduced with T1-T2 similarity. Our results are consistent with the predictions and the results of the Di Lollo et al. (2005) experiment, where AB was reduced for targets within the same category. Alternatively, increase in T1-T2 similarity may increase the stability of working memory representations and thereby reduce interference. The magnitude of the AB is therefore expected to decrease with an increase of T1-T2 similarity, as was revealed in our experiment.

Our results are inconsistent with the results of Awh et al. (2004), whose study indicates that no AB interference was revealed when a digit target preceded a face target, which is related to the different types of processing required in this case, and a severe AB was revealed with the same types of processing. According to the multiple channels for configural and featural processing hypothesis, the severity of the AB depends on the extent to which T1 and T2 processing assumes the same mechanisms. Our concern is that in the Awh et al. (2004) experiments, the manipulation was related to the type of processing (T1 and T2 could be face or digit), whereas our experiments (as well as the priming and categorical similarity studies) manipulated the perceptual similarity within the same type of processing (both T1 and T2 were geometric shapes, the manipulation referred to the number of shared features).

Whereas similarity by the one task-irrelevant feature (size) or by two features (one task-relevant and one task-irrelevant) reduced the AB, similarity on task-relevant feature (shape) produced the attentional blink with no lag 1 sparing. A possible explanation might be the response bias, but the T1 accuracy reveals it is not the case here. Another might be interference: whereas the similarity of task-irrelevant features would increase the stability of working memory representations, similarity of task-relevant features (especially on lag 1, when T1 and T2 are processed simultaneously) would increase the interference. This is actually consistent with the difference in results observed for T1-T2 similarity: similarity on the level of target features enhanced the T2 accuracy, whereas the same type of processing required from T1 and T2 increased the magnitude of the AB.

Another point worthy of mention is that the observed attentional blink in Experiment 2 was smaller across conditions even though it is a very similar experiment to that of Experiment 1. This comes from the frequency of task-relevant conditions not properly balanced in Experiment 1, but balanced in Experiment 2. Shared size feature condition included 120 trials, and shared shape feature condition included 40 trials. Shared size feature condition also revealed better accuracy as compared with shared shape feature condition (according to additional *post hoc* analysis for the one shared-feature condition, presented on Figure 4), at that point, the observed attentional blink in Experiment 1 was larger across conditions as compared with Experiment 2.

## Experiment 3

The key finding of Experiment 2 does not match with Awh et al. (2004) or Sy and Giesbrecht (2009) experiments. One of the possible explanations might be the consequence of mixing the different types of trials together that may engender a strategy that promotes a holistic encoding that might benefit from similarity. The previous studies that have shown more severe ABs with increased similarity have tended to block the conditions. Moreover, having extended lags would allow one to determine the severity of the full AB. Considering these two issues, a new experiment was conducted. Modifications included a blocked design and extended lags.

Due to the COVID-19 pandemic and in order to take care of the health of the participants, the experiment was run online using Pavlovia software. In order to keep the data reliability, sample size was increased. Also, the same screen resolution and refresh rate was set for all participants.

The invitation to participate in the experiment was posted on Russian popular social network page, dedicated to jokes about cognitive science – “Cognitive Partymaker.”^3^ Volunteers were instructed that participants of the experiment can participate in a lottery with the opportunity to win 1,000 rubles (around $14) or the set of stickers. Volunteers had to fill out the Google form, indicating their age, gender, possible neurological and psychiatric difficulties, and contacts and were later instructed to set the refresh rate and screen resolution of their monitors and the link for the experiment.

### Method

#### Participants

Sample included 48 participants (34 females, 14 males). The age varied from 18 to 25 years old (M = 21.625, SD = 1.963). All participants were native Russian speakers, naive to experimental hypotheses, and reported normal or corrected-to-normal visual acuity. Informed consent was obtained from all subjects.

#### Stimuli and Procedure

Stimuli and procedure were similar to Experiment 2, except for the number of lags and blocked design. There were six lags and four experimental blocks (one block for each condition). Overall, there were 576 trials, distributed equally between six lag conditions and between four shared-feature factor conditions: two shared features (shape and size), shared size feature, shared shape feature, and 0 shared features. The order of block presentation was counterbalanced across subjects.

Experiment was conducted online, using the Pavlovia.org webpage. All the participants had the same screen resolution and refresh rate on their monitors (screen resolution, 1,920 × 1,080; refresh rate, 60 Hz). Participants’ responses were registered with a standard keyboard.

### Results

Data analysis followed the analysis proposed for Experiment 2. The factors included the number of shared features (levels: two shared features, same shape, same size, and 0 shared features) and lag (lags 1–6). Moreover, a separate ANOVA for lag factor (levels: lags 1, 2, 3, 4, 5, and 6) was conducted within each level of the shared-feature factor in order to precisely test the AB effect for each condition (due to the significant lag × shared-feature interaction). Pairwise comparisons were made with Bonferroni corrections; corrections were applied within each analysis.

#### T1 Performance

ANOVA revealed the insignificant effect of the number of shared-feature factor [*F* (2, 109) = 2.58, *p* = 0.072, *ηp*^2^ = 0.052]. The lag factor was also significant [*F* (4, 205) = 6.77, *p* < 0.001, *ηp*^2^ = 0.126], as well as the interaction of these factors [*F* (9, 445) = 4.737, *p* < 0.001, *ηp*^2^ = 0.092]. According to pairwise comparisons, one shared-feature condition (size) accuracy was higher as compared with one shared-feature condition (shape; *t* = 3.146, *p* = 0.017, *d* = 0.318). According to pairwise comparisons for the lag factor, lag 1 accuracy was lower as compared with lag 2 (*t* = −4.523, *p* = 0.001, *d* = −0.239) and lag 3 (*t* = −3.818, *p* = 0.006, *d* = −0.239). Lag 2 accuracy was higher as compared with lag 5 (*t* = 3.820, *p* = 0.006, *d* = 0.227) and lag 6 (*t* = 3.205, *p* = 0.037, *d* = 0.208). Lag 3 accuracy was higher as compared with lag 5 (*t* = 4.218, *p* = 0.002, *d* = 0.227) and lag 6 (*t* = 3.179, *p* = 0.039, *d* = 0.207).

ANOVA within the two shared-feature condition revealed the significant impact of the lag factor [*F* (5, 235) = 5.26; *p* < 0.001; *ηp*^2^ = 0.101]. According to pairwise comparisons, lag 1 accuracy was higher as compared with lag 2 (*t* = −0.454, *p* = 0.025, *d* = −0.049), lag 3 (*t* = 1.137, *p* = 0.008, *d* = 0.123), lag 4 (*t* = 1.917, *p* = 0.001, *d* = 0.289), and lag 5 (*t* = 1.453, *p* = 0.016, *d* = 0.201).

ANOVA within the shared shape feature condition revealed significant impact of lag factor [*F* (4, 197) = 8.43; *p* < 0.001; *ηp*^2^ = 0.152]. According to pairwise comparisons, lag 1 accuracy was higher as compared with lag 2 (*t* = 1.663, *p* < 0.001, *d* = 0.167) and lag 4 (*t* = 0.903, *p* = 0.001, *d* = 0.084). Lag 1 accuracy was lower as compared with lag 3 (*t* = −0.278, *p* = 0.001, *d* = −0.036). Lag 2 accuracy was higher as compared with lag 5 (*t* = 1.985, *p* = 0.025, *d* = 0.232) and lower as compared to lag 3 (*t* = −2.186, *p* = 0.001, *d* = −0.246).

ANOVA within the shared size feature condition revealed the significant impact of lag factor [*F* (4, 170) = 4.226; *p* = 0.004; *ηp*^2^ = 0.083]. According to pairwise comparisons, lag 5 accuracy was lower as compared with lag 6 (*t* = −0.638, *p* = 0.022, *d* = −0.062) and lag 3 (*t* = −2.287, *p* = 0.009, *d* = −0.216).

ANOVA within the 0 shared-feature condition revealed a significant impact of the lag factor [*F* (4, 175) = 3.658; *p* = 0.008; *ηp*^2^ = 0.072]. According to pairwise comparisons, lag 2 accuracy was higher as compared with lag 6 (*t* = 0.677, *p* = 0.008, *d* = 0.045).

**Figure 7:**
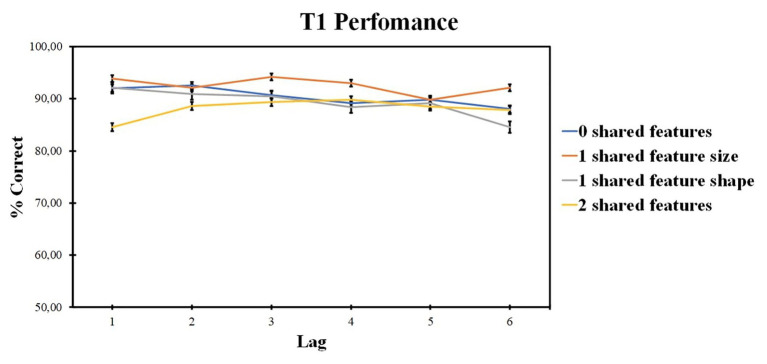
Results of experiment 3: T1 identification accuracy. Error bars show the standard errors of the mean.

Those results are presented in Figure 7.

#### T2|T1 Performance

ANOVA revealed no significant effect for the number of shared-feature factor [*F* (3, 141) = 1.16, *p* = 0.326, *ηp*^2^ = 0.024, BF_10_ = 0.471]. The lag factor was significant [*F* (4, 18) = 5.49, *p* < 0.001, *ηp*^2^ = 0.390, BF_10_ = 1.603е + 12]. The interaction was also significant [*F* (9, 413) = 3.55, *p* < 0.001, *ηp*^2^ = 0.070]. According to pairwise comparisons for the lag factor, lag 1 accuracy was lower as compared with lag 3 (*t* = −3.7, *p* = 0.008, *d* = −0.274), lag 4 (*t* = −4.204, *p* = 0.002, *d* = −0.34), lag 5 (*t* = −10.388, *p* < 0.001, *d* = −0.808), and lag 6 (*t* = −6.578, *p* < 0.001, *d* = −0.602). Lag 2 accuracy was lower as compared with lag 3 (*t* = −3.54, *p* = 0.014, *d* = −0.263), lag 4 (*t* = −4.511, *p* = 0.001, *d* = −0.329), lag 5 (*t* = −8.961, *p* < 0.001, *d* = −0.798), and lag 6 (*t* = −6.558, *p* < 0.001, *d* = −0.592). Lag 3 accuracy was lower as compared with lag 5 (*t* = −6.252, *p* < 0.001, *d* = −0.49) and lag 6 (*t* = −3.368, *p* = 0.023, *d* = −0.312). Lag 5 accuracy was higher as compared with lag 4 (*t* = 6.113, *p* < 0.001, *d* = 0.517) and lag 6 (*t* = 3.836, *p* = 0.006, *d* = 0.209).

ANOVA within the 0 shared-feature condition revealed the significant impact of the lag factor [*F* (3, 163) = 16.61; *p* < 0.001; *ηp*^2^ = 0.261]. According to pairwise comparisons, lag 1 accuracy was lower as compared with lag 5 (*t* = −5.425, *p* < 0.001, *d* = −0.779) and lag 6 (*t* = −4.944, *p* < 0.001, *d* = −0.608). Lag 2 accuracy was lower as compared with lag 3 (*t* = −3.788, *p* = 0.006, *d* = −0.529), lag 5 (*t* = −7.705, *p* < 0.001, *d* = −0.96), and lag 6 (*t* = −7.748, *p* < 0.001, *d* = −0.798). Lag 3 accuracy was lower as compared with lag 5 (*t* = −3.57, *p* = 0.013, *d* = −0.45). Lag 4 accuracy was lower as compared with lag 5 (*t* = −5.238, *p* < 0.001, *d* = 0.76) and lag 6 (*t* = −5.398, *p* < 0.001, *d* = 0.596).

ANOVA within the shared size feature condition revealed significant impact of lag factor [*F* (3, 158) = 5.78; *p* = 0.001; *ηp*^2^ = 0.109]. According to pairwise comparisons, lag 1 accuracy was lower as compared with lag 4 (*t* = −3.807, *p* = 0.006, *d* = −0.144) and lag 5 (*t* = −4.494, *p* = 0.001, *d* = −0.422). Lag 2 accuracy was lower as compared with lag 4 (*t* = −4.814, *p* = 0.006, *d* = −0.633) and lag 5 (*t* = −3.661, *p* = 0.01, *d* = −0.514).

ANOVA within the shared shape feature condition revealed the significant impact of lag factor [*F* (4, 197) = 10.756; *p* < 0.001; *ηp*^2^ = 0.186]. According to pairwise comparisons, lag 1 accuracy was lower as compared with lag 5 (*t* = −6.915, *p* < 0.001, *d* = −0.8) and lag 6 (*t* = −3.326, *p* = 0.026, *d* = −0.544). Lag 5 accuracy was higher as compared with lag 2 (*t* = 6.771, *p* < 0.001, *d* = 0.657), lag 3 (*t* = 4.866, *p* < 0.001, *d* = 0.536), and lag 4 (*t* = 5.524, *p* < 0.001, *d* = 0.655).

ANOVA within the two shared-feature condition revealed no significant impact of the lag factor [*F* (3, 156) = 3.121; *p* = 0.084; *ηp*^2^ = 0.062].

**Figure 8:**
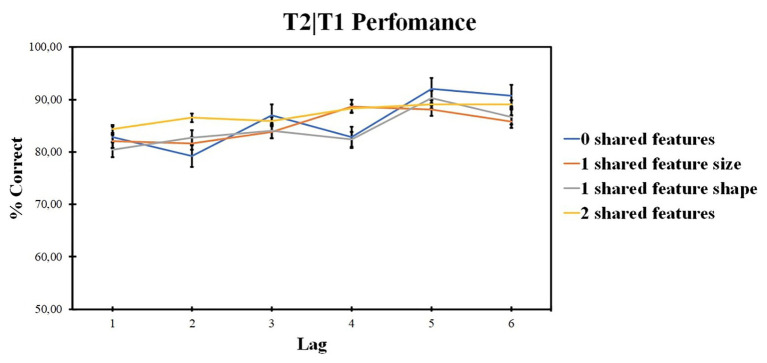
Results of experiment 3: T2 identification accuracy for trials on which T1 was correctly identified (T2|T1). Error bars show the standard errors of the mean.

Those results are presented in Figure 8.

### Discussion

Overall performance in Experiment 3 was better as compared with Experiment 1 and Experiment 2, which could be related to decreased task difficulty in blocked design as well as to increased subjects vigilance, as the participants were instructed that only the ones who provided reliable data would participate in the lottery for money. T1 performance for the task-relevant feature condition was reduced, revealing the issues of blocked design that allows the participants to use strategy more as compared with randomized trial design, especially when T1 and T2 are perceptually similar.

No attentional blink was observed for two shared-feature condition. For shared size feature condition, same shape feature condition and no shared-feature condition attentional blink without lag 1 sparing were observed.

The pattern observed for no shared-feature condition is similar to Experiment 2, except no lag 1 sparing observed for blocked design. Visser et al. (1999) suggested that lag 1 sparing is absent when observers have to substantially alter their attentional set from T1 to T2 (e.g., switches in target task or switches in target category are required). The presentation of T1 initiates an attentional gate that allows the item presented directly after T1 to access the attentional resources, if it is broadly similar to T1 so that it can pass through the same input filter. With randomized trials design (Experiment 2), T2 could be expected to be similar to T1, so the holistic encoding strategy could be applied, whereas with blocked design (Experiment 3), this was not possible. At that point, lag 1 sparing is absent for blocked design for perceptually dissimilar targets.

The pattern observed for T2 detection in two shared-feature condition is equal to observed in Experiment 2 and consistent with the initial predictions. However, the effect size was less as compared with Experiment 2, so T2 processing did not benefit from similarity in blocked design as much as in mixed-trial design. What is also worth mentioning is T1 performance that increased with the lag. At that point, blocked design assumed more interference between perceptually similar T1 and T2 on earlier lags (which is supported by T2 data in one shared-feature conditions), or more response bias when T1 and T2 followed one after another.

Similarity on task-relevant feature (shape) produced the attentional blink with no lag 1 sparing, as in Experiment 2, supporting the idea of T1-T2 interference when the targets are perceptually similar on task-relevant feature. However, similarity on task-irrelevant feature (size) produced the attentional blink as well, unlike Experiment 1. At that point, mixed-trial design could engender a strategy that promotes a holistic encoding that might benefit from similarity, whereas blocked design did not have this opportunity. However, recovery from the blink was observed earlier for targets similar on task-irrelevant feature, which considers that the task-relevant feature similarity would produce the more severe T2 processing deficit, as observed in earlier studies (e.g., Sy and Giesbrecht, 2009).

## Conclusion

Three experiments investigated the role of target-target similarity in AB. In all experiments, we manipulated the number of shared features between T1 and T2. Conditions included 0 shared features, one shared feature (task-relevant – shape or task-irrelevant – size), and two shared features. In Experiment 1, we balanced different types of targets, whereas in Experiment 2, we balanced the number of task-relevant and task-irrelevant trials, so Experiment 2 provided more reliable results in terms of response bias. Experiment 3 was similar to Experiment 2 except blocked design was used.

In Experiment 2, AB with lag 1 sparing was observed for the 0 shared-feature condition, and AB with no lag 1 sparing was observed for the one task-relevant shared-feature condition. No AB was observed for the one task-irrelevant shared-feature condition and for the two shared-feature condition. In Experiment 3, similarity effects were significantly lower as compared with Experiment 2 and observed only for two shared-feature condition.

The reduction of AB for the two shared-feature condition (and for the one task-irrelevant shared-feature condition in Experiment 2) is consistent with the predictions of interference in working memory account, whereas multiple channels for configural and featural processing model would predict the opposite results. These results are also consistent with the previous findings for change detection tasks (Lin and Luck, 2009) and dual-target visual search tasks (Gorbunova, 2017), as well as the AB studies of priming (e.g., Maki et al., 1997) and categorical similarity (Taylor and Hamm, 1997). No lag 1 sparing observed for the task-relevant feature condition is congruent with the predictions of multiple channels for the configural and featural processing model, and with the results of studies that manipulated the similarity as the type of processing (Awh et al., 2004; Sy and Giesbrecht, 2009). The point here might be different kinds of similarity having opposite effects: similarity on the level of the task would increase the amount of resources required for T2 processing, whereas the similarity on the level of target features would increase the stability of working memory representations. However, this benefit from similarity was present more for mixed-trial design as compared with blocked design. Blocked design could engender a strategy that promotes a holistic encoding that might benefit from similarity. What is worth mentioning is that the previous studies that have shown more severe AB deficit with increased similarity usually used blocked design.

In addition, considering the repetition blindness studies, our results should also be discussed in terms of RB. RB is proposed to reflect a failure to individuate separate tokens for two repeated events (Kanwisher, 1987), whereas AB is the overall visual-type discriminability along the feature dimension to be reported (Chun, 1997). The effect observed in our study is unlikely to be RB. First of all, the task in our experiment was to report the shape of a predefined target, as in AB studies, but not to report the items in the stream, as in RB paradigm. Moreover, T1 and T2 in all conditions were different in color, whereas different T1 and T2 color should have alleviated RB. According to previous studies (e.g., Chun, 1997), RB magnitude is expected to be reduced when the perceptual difference between T1 and T2 is increased – the effect which is opposite to that observed in our study. The crucial difference between the study reported here and most of RB studies is that T1 and T2 were always defined as different spatiotemporal tokens (because they had different color), so increasing the perceptual similarity between T1 and T2 in terms of shared features did not affect token individuation.

## Data Availability Statement

The raw data supporting the conclusions of this article will be made available by the authors, without undue reservation.

## Ethics Statement

Ethical review and approval was not required for the study on human participants in accordance with the local legislation and institutional requirements. The patients/participants provided their written informed consent to participate in this study.

## Author Contributions

EG: originator of the concept, experimental planning, and manuscript preparation. IM: experimental planning, programming, data collection, data analysis, and manuscript preparation. All authors contributed to the article and approved the submitted version.

### Conflict of Interest

The authors declare that the research was conducted in the absence of any commercial or financial relationships that could be construed as a potential conflict of interest.

## Footnotes

^1^Different numbers of trials for each condition were used because of different numbers of variations for shapes and sizes (four shapes, two sizes) in order to make all possible feature combinations equally present.

^2^The pilot study with a single task (only T2 detection) revealed no differences for T2 detection for different lags for the chosen timing.

^3^https://vk.com/cogparty
